# Polymeric Flexible Immunosensor Based on Piezoresistive Micro-Cantilever with PEDOT/PSS Conductive Layer

**DOI:** 10.3390/s18020451

**Published:** 2018-02-03

**Authors:** Rui Zhao, Ying Sun

**Affiliations:** School of Instrument and Electronics, North University of China, Taiyuan 030051, China; sunyy92@163.com

**Keywords:** flexible micro-cantilever, PEDOT/PSS, polymeric sensor, immunoassay

## Abstract

In this paper, a fully polymeric micro-cantilever with the surface passivation layer of parylene-C and the strain resistor of poly(3,4-ethylenedioxythiophene)/poly (styrene sulfonate) (PEDOT/PSS) was proposed and demonstrated for immunoassays. By optimizing the design and fabrication of the polymeric micro-cantilever, a square resistance of 220 Ω/□ for PEDOT/PSS conductive layer have been obtained. The experimental spring constant and the deflection sensitivity were measured to be 0.017 N/m and 8.59 × 10^−7^ nm^−1^, respectively. The biological sensing performances of polymeric micro-cantilever were investigated by the immunoassay for human immunoglobulin G (IgG). The immunosensor was experimentally demonstrated to have a linear behavior for the detection of IgG within the concentrations of 10~100 ng/mL with a limit of detection (LOD) of 10 ng/mL. The experimental results indicate that the proposed polymeric flexible conductive layer-based sensors are capable of detecting trace biological substances.

## 1. Introduction

The microminiaturization sensing devices based on the micro-electromechanical system (MEMS) technique have been widely used in the applications of biological and chemical detections [1,2]. Micro-cantilevers, beam structures with one end fixed on the substrate and with a thickness in several micrometer range, exhibit various attractive properties and have been successfully used as biochemical detection platforms due to their small size and ultra-high sensitivity [3,4,5]. When an external force was applied by pressing the free end of micro-cantilever, a mechanical deformation will be generated and then converted into readable optical or electrical signals. The optical domain method provides ultra-high resolution measurement and linear response for the induced deformation, but the bulk of the instrument and the requirement of technical expertise for operating equipment restrict the applications for portable microminiaturization sensing devices [6,7].

Therefore, some researchers have turned attention to employing a micro-cantilever with an embedded piezoresistor to convert mechanical deformation into a resistance change [8,9,10]. When a micro-cantilever with piezoresistor located at an appropriate region is slightly deformable, the change in the piezoresistor reflects the extent of the deflection. This change can be measured by a Wheatstone bridge supplied with DC bias voltage. A typical piezoresistor is fabricated by boron-doped polysilicon or monocrystalline silicon, which has been used for lots of sensors extensively [11,12,13]. However, low sensitivity is a disadvantage of silicon-based piezoresistive micro-cantilevers due to the high Young’s modulus of silicon materials. It is demonstrated that the sensitivity can be improved by using polymer materials with a lower Young’s modulus to fabricate micro-cantilever [14,15,16]. Unfortunately, SU-8 has been demonstrated to be a good polymer material for humidity sensing due to its hygroscopic nature [17], so the output of piezoresistive micro-cantilever based on SU-8 is unstable when exposed to liquid phase for an extended period of time. Parylene-C, which has a similar Young’s modulus to SU-8, has been indicated to be an encapsulation and passivation layer for increasing the stability of piezoresistive micro-cantilevers [18].

Recently, the flexible microminiaturization sensors provide another unique opportunity in the application of detecting biological substances with the emergence of organic electronics [19,20,21,22,23]. Poly(3,4-ethylenedioxythiophene)/poly (styrene sulfonate) (PEDOT/PSS), a typical flexible conductive polymer, is highly interesting due to its high conductivity, thermal stability, and low cost processing ability [24,25]. In particular, the usability of PEDOT/PSS conductive layer has been reported for electromechanical sensor based on the piezoresistive effect [26,27,28,29,30].

In this work, a fully polymeric micro-cantilever was designed and fabricated by using PEDOT/PSS conductive layer as piezoresistor and parylene-C as the surface passivation layer. The square resistances of PEDOT/PSS were characterized and optimized to be 220 Ω/□. The fabricated polymeric micro-cantilever was experimentally demonstrated with high sensitivity. An immunosensor was developed by the functionalization of the micro-cantilevers, which can be used to trap human immunoglobulin G (IgG) molecules in phosphate buffered saline (PBS) with the limit of detection (LOD) of 10 ng/mL.

## 2. Micro-Cantilever Design and Fabrication

**Figure 1:**
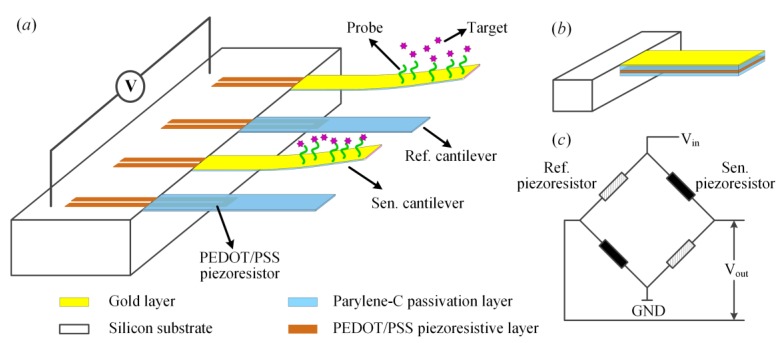
The schematics of (**a**) a polymeric micro-cantilever-based sensor; (**b**) a sensing piezoresistive micro-cantilever based on PEDOT/PSS; and (**c**) arrangement of two pairs of polymeric micro-cantilevers as a Wheatstone bridge for signal readout. The length, width, and thickness of polymeric micro-cantilever are 150, 75, and 2 μm, respectively. The leg dimensions of PEDOT/PSS based piezoresistor are 80 × 8 μm.

The proposed polymeric piezoresistive micro-cantilever is operated for the detection of surface stress change by measuring the static deflection of micro-cantilever, which was designed to be rectangular in the dimensions of 150 × 75 × 2 μm with a PEDOT/PSS piezoresistor embedded in it. The flexible sensor formed by four identical micro-cantilevers was configured by a Wheatstone bridge with two PEDOT/PSS-based piezoresistors placed on sensing micro-cantilevers and two on reference micro-cantilevers, as shown in Figure 1a. This design has a significant suppression effect on mechanical and environmental noise. Figure 1b shows the composition of sensing micro-cantilever, including top and bottom parylene-C passivation layers, a PEDOT/PSS piezoresistive layer encapsulated by two parylene-C passivation layers and a 5/40 nm Ti/Au modified layer. Different from the sensing micro-cantilever, there is no Ti/Au modified layer on the top surface of the reference micro-cantilever. The piezoresistors were designed to be four-fold structure with a single piezoresistor leg dimensions of 80 × 8 μm.

During the operation, the top surface of sensing micro-cantilever is modified by immobilization of specific probes to trap the targets in solution, the binding process between probes and targets will induce a surface stress on the top surface and result in a deformation for the sensing micro-cantilever. Different from the sensing micro-cantilever, there is no surface stress to be induced for reference micro-cantilevers due to the absence of gold film. The relation between the vertical deflection (Δ*d*) and the surface stress change (Δ*σ*) can be written as [31]

(1) $$\frac{\Delta d}{\Delta \sigma }=\frac{3\cdot (1-\nu )\cdot l^{2}}{E\cdot t^{2}}$$

where *ν* and *E* are Poisson’s ratio and Young’s modulus of micro-cantilever materials, *l* and *t* are the length and thickness of the micro-cantilever. Figure 1c is the schematic of micro-cantilever array arrangement as a Wheatstone bridge circuit for signal readout. The differential output of Wheatstone bridge, *V_out_*, can be calculated by *V_out_* = 1/2·*V_in_*·Δ*R*/*R*, where *V_in_* represents the DC voltage supplied across the Wheatstone bridge, Δ*R*/*R* reflects the relative resistance change of the piezoresistor.

**Figure 2:**
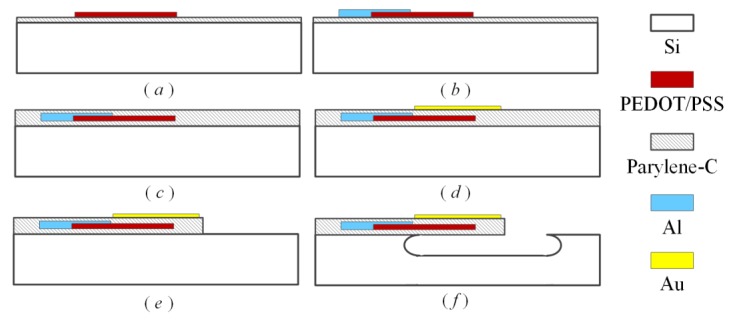
The fabrication processes of the proposed flexible micro-cantilever with PEDOT/PSS piezoresistor.

**Figure 3:**
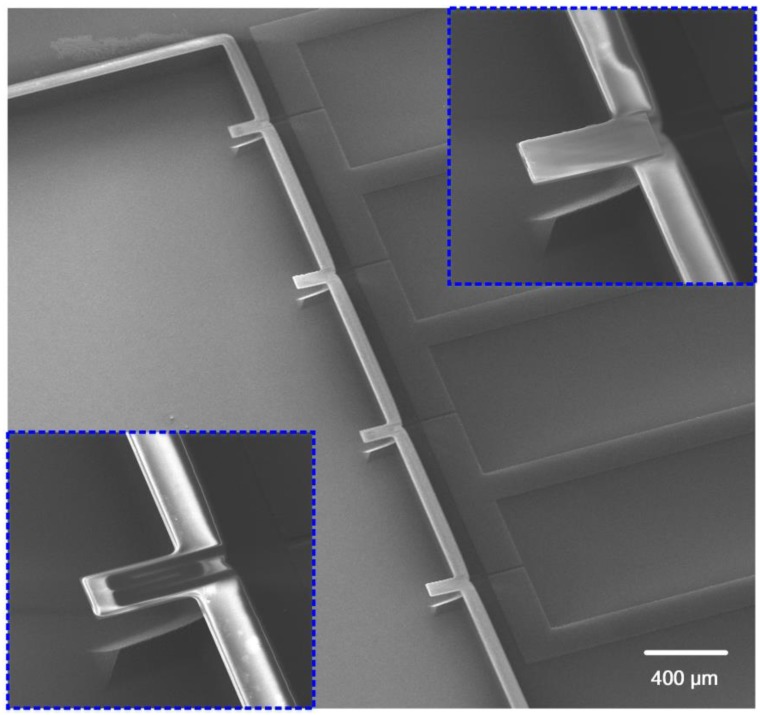
The SEM photo of fabricated polymeric micro-cantilever array (Top-right and left insets present a sensing cantilever and a reference cantilever, respectively).

The fabrication processes of the proposed polymeric cantilever start from single polished silicon wafers, which are sketched in Figure 2 and described as follows: (a) A parylene-C passivation layer with the thickness of 1 μm was deposited by chemical vapor deposition (CVD) technology. After that, a PEDOT/PSS conductive layer was spin-coated and patterned by oxygen plasma to form piezoresistors. (b) An 700 nm aluminum layer was sputtered and patterned to realize the electrical wires and the pad areas. (c) The other parylene-C layer was deposited as passivation layer to fully encapsulate PEDOT/PSS piezoresistors and electrical wires together with the bottom parylene-C passivation layer. (d) A Ti/Au modified layer with a thickness of 5/40 nm was sequentially sputtered by physical vapor deposition technology and patterned on the top surface of the sensing micro-cantilevers by wet etching process. (e) The micro-cantilever areas were patterned and etched by oxygen plasma together with the contact holes being opened. (f) The polymeric micro-cantilevers were released by a hybrid process of SF_6_ anisotropic and isotropic dry etching. A scanning electronic microscopy (SEM) photo of the fabricated polymeric micro-cantilever array is shown in Figure 3, where the top-right insert shows a polymeric sensing micro-cantilever and the left insert exhibits a polymeric reference micro-cantilever.

It is worth pointing out that a series of attempts were implemented to optimize the electrical properties of PEDOT/PSS conductive layer and the adhesivity on silicon substrate. Firstly, silane coupling agent, *γ*-glycidoxy propyltrimethoxy silane (*γ*-GPS), was uniformly mixed with the intrinsic PEDOT/PSS solution (Clevios PH 1000, Leverkusen, Germany) to increase the adhesivity on the substrate [32]. After that, dimethyl sulfoxide (DMSO) as conductive enhancer was added to decrease the square resistance of PEDOT/PSS conductive layer [33]. Subsequently, isopropyl alcohol (IPA) was mixed to improve the wettability of PEDOT/PSS solution and the uniformity of PEDOT/PSS conductive layer on the substrate [34]. The square resistance of PEDOT/PSS conductive layer was optimized to be 220 Ω/□ (Standard deviation=7 Ω/□, *n* = 20) by using the mixing solution of PEDOT/PSS, *γ*-GPS, DMSO, and IPA (V_γ-GPS_:V_DMSO_:V_IPA_:V_PEDOT/PSS_ = 0.5:5:50:100 in volume).

## 3. Characteristics of the Polymeric Micro-Cantilever

**Figure 4:**
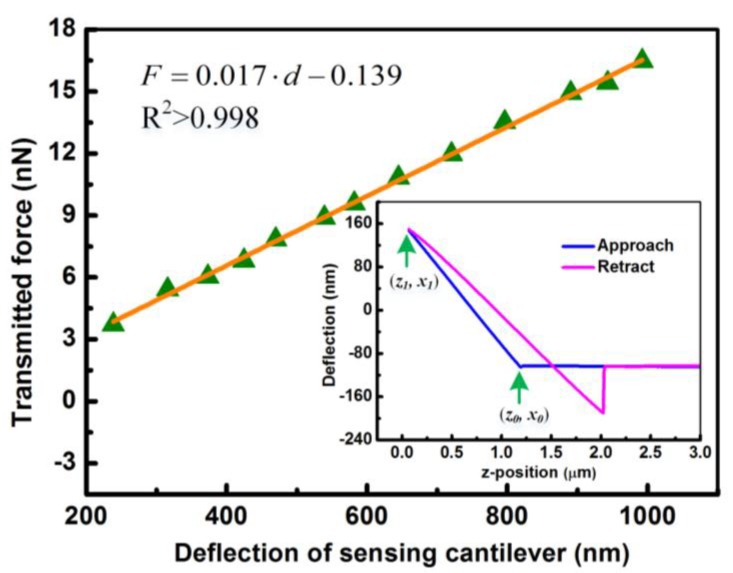
Transmitted force versus deflection for measuring the stiffness of the polymeric sensing micro-cantilever. Insert plots AFM force curves on the polymeric sensing micro-cantilever. The approach curve was used to calibrate the stiffness of polymeric sensing micro-cantilever.

The expression of stiffness for the rectangular micro-cantilever can be written as [35]

(2) $$k=\frac{E\cdot w\cdot t^{3}}{4\cdot l^{3}}$$

where *w* represents the micro-cantilever width. It is obvious that the geometries of the micro-cantilever and the Young’s modulus are of appreciable contributions to the stiffness. To measure the stiffness of the sensing micro-cantilever, a commercial AFM (Nanoworld, PNP-DB, Neuchâtel, Switzerland) with a stiffness of 0.06 N/m (*k*_AFM_ = 0.06 N/m) was acted on the free end of sensing micro-cantilever with a transmitted force. The transmitted force *F* is expressed as [36]

(3) $$F=k_{AFM}\cdot (x_{1}-x_{0})$$

where *x*_0_ and *x*_1_ are the initial and final deformations of the commercial AFM detected by a photodiode (see inset of Figure 4). The deformation of the sensing micro-cantilever, *d*, is estimated by the difference of the net deflection of the AFM and the total *z*-travel, as expressed by [37]

(4) $$d=\left|z_{1}-z_{0}\right|-\left|x_{1}-x_{0}\right|$$

Figure 4 shows the relations between the transmitted force *F* and the deflection *d*, in which the calibrated stiffness for the polymeric sensing micro-cantilever is found to be 0.017 N/m with the goodness of fit (R^2^-value) greater than 0.998 by the slope of the linear fit of transmitted force and the deflection of sensing micro-cantilever. These results show that the fabricated polymeric micro-cantilever has a linear response.

**Figure 5:**
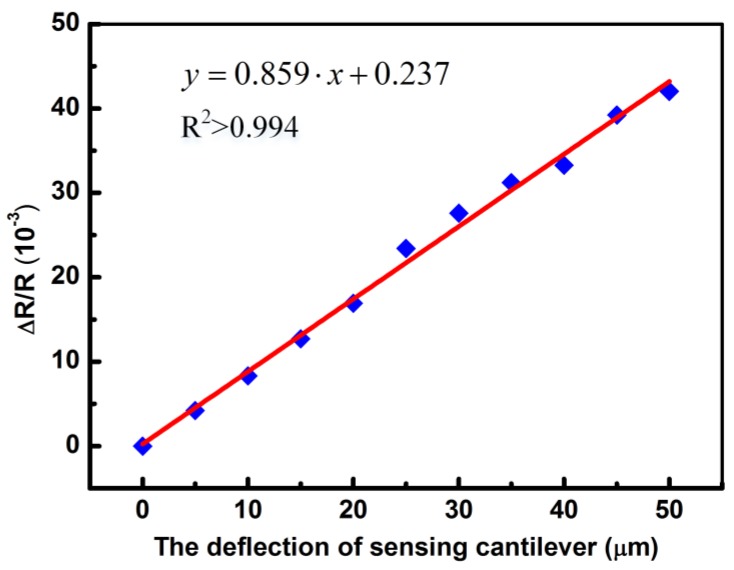
The relative resistance changes of PEDOT/PSS piezoresistor measured for net deflection of 50 μm in 5 μm steps, the deflection sensitivity was acquired by the slope of the fitted line (R^2^ > 0.994).

The sensitivity of the fabricated polymeric micro-cantilever was acquired by measuring the relative resistance change (Δ*R*/*R*) of PEDOT/PSS based piezoresistor, which was recorded by an Agilent 34401A 6½ digital multimeter. A precision stage was used to measure the vertical deflection of the free end for polymeric micro-cantilever by controlling in 5 μm steps. Figure 5 shows the relation between (Δ*R*/*R*) and Δ*d* of the fabricated polymeric micro-cantilever, where a deflection sensitivity (Δ*d*^−1^∙Δ*R*/*R*) of 8.59 × 10^−7^ nm^−1^ was extracted by the slope of the fitted line. Compared with several works previously reported [15,38,39], the polymeric piezoresistive micro-cantilever described here shows smaller deflection sensitivity, which can be attributed to the differences of the gauge factor and the dimensions of the micro-cantilever and the piezoresitor [11].

## 4. Immunoassay Experiments

### 4.1. Reagents and Materials

During experiments, PBS, 3,3′-dithiopropionic acid (DDPA), N-hydroxy succinimide (NHS), 1-ethyl-3-(3-dimethylaminopropyl) carbodiimide hydrochloride (EDC), streptavidin, ethanolamine, bovine serum albumin (BSA) were received from Sigma-Aldrich Co. LLC. (Beijing, China). Biotinylated rabbit anti-human IgG polyclonal antibody (bio-PcAb against IgG) and IgG were purchased from Beijing Biosynthesis Biotechnology Co., Ltd. (Beijing, China). Acetone (analytical reagent) and alcohol (analytical reagent) were obtained from Suzhou Crystal Clear Chemical Co., Ltd. (Suzhou, China). The bio-PcAb against IgG was serial diluted with PBS (0.01 M, pH 7.4) to obtain the solution with the concentration of 10 μg/mL. The IgG solutions of different concentrations (0.2~2 μg/mL) were obtained via serial dilution of an original IgG solution. Subsequently, all IgG solutions and bio-PcAb against IgG solutions were frozen at −18 °C for the contrast tests and recovery experiments. All aqueous solutions were prepared with double-distilled water.

### 4.2. Surface Functionalization

**Figure 6:**
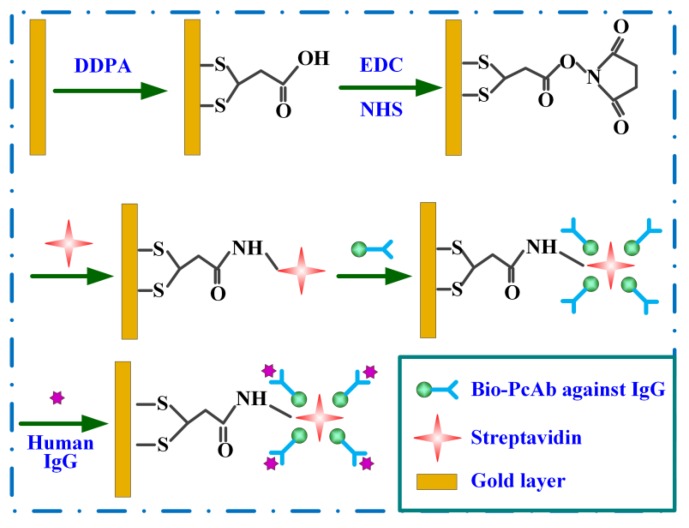
Schematic diagram of the functionalization processes on the polymeric sensing micro-cantilevers and IgG molecules binding with bio-PcAb against IgG.

Prior to functionalization of the polymeric sensors, they were pretreated with oxygen plasma (250 W, 20 sccm) for 30 s to remove the denaturalized layer of photoresist and organic contamination. The polymeric micro-cantilevers were then cleaned twice with acetone for 20 min followed by alcohol rinse to remove the organic contamination thoroughly. Finally, the polymeric micro-cantilevers were rinsed repetitiously by double-distilled water and dried in nitrogen atmosphere. Figure 6 describes the functionalization processes for the polymeric sensors. Firstly, the polymeric micro-cantilevers were incubated in DDPA (5 mg/mL) for 30 min to self-assemble a carboxyl monolayer by Au-S covalent. After that, the polymeric micro-cantilevers were immersed into EDC/NHS (5 mg/mL, 3:1 in volume) mixing solution for 30 min, which will form a succinimide esters layer produced by the reaction of carboxyl group and EDC/NHS. After the streptavidin (0.1 mg/mL) tethered to the amine-reactive NHS ester, the remaining carboxyl group on gold film were inactivated by adding ethanolamine (1 M). The penultimate step was that the unbound streptavidin and ethanolamine molecules were eluted by PBS. Finally, the polymeric micro-cantilevers were incubated in the solution of bio-PcAb against IgG with the concentration of 10 μg/mL to immobilize the probe molecules by the interactions of the bio-PcAb against IgG and streptavidin. The functionalization of polymeric micro-cantilevers was finished after a multiple rinse to remove the unbound probes by PBS. All the procedures were performed at room temperature.

### 4.3. IgG Detection in Buffer

During immunoassay, different sensors functionalized with the same process were used for detecting IgG with the different concentrations. To monitor the response of the polymeric sensors, the sensor chip was fixed on a printed circuit board (PCB). The wire bonding technology was adopted to realize the electrical connection between chip and PCB. During immunoassay experiments, the sensors were put in a container with uncirculated PBS solution (0.01 M, pH 7.4) of 400 μL and a DC voltage of 3 V was supplied to the Wheatstone bridge. The output voltage was introduced in an Agilent 34401A digital multimeter and recorded by a PC every 2 s. After acquiring a stable baseline, 20 μL IgG solutions with different concentrations were dropped into the container with PBS separately, the corresponding concentrations of IgG are 10 ng/mL~100 ng/mL.

**Figure 7:**
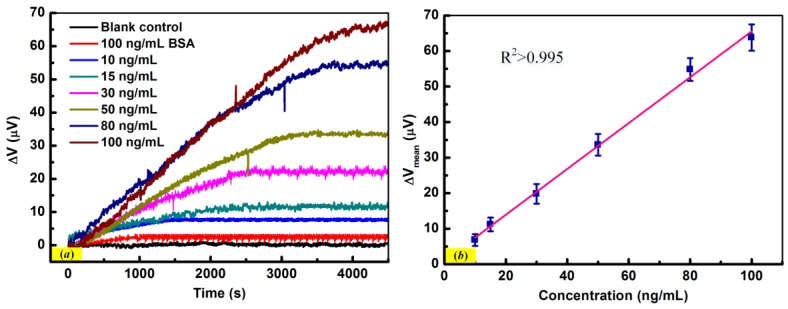
(**a**) The response curves of immunosensors detecting IgG with the concentrations of 10, 15, 30, 50, 80, and 100 ng/mL, respectively. (**b**) The steady-state output voltage of immunosensors versus the concentrations of IgG in PBS, which shows a linear relation with the goodness of fit (R^2^-value) above 0.995.

Figure 7a plots the responses caused by the interaction of bio-PcAb against IgG and IgG on the sensing micro-cantilever surface. It is obvious that the sensors showed steady-state responses for all immunoassay experiments, while the time spent to achieve the steady-state is shorter and the response amplitude is smaller for the lower concentration IgG. This experimental phenomenon can be attributed to the initial non-equilibrium of the system [40]. The reproducibility of the proposed polymeric immunosensor was evaluated, whose experiments were performed for five times with five identical functionalized sensors separately. The response changes for the six additions are 64 ± 4, 55 ± 3, 34 ± 3, 20 ± 3, 11 ± 2, and 7 ± 2 μV, respectively (*n* = 5). A negative control experiment was implemented by using 100 ng/mL BSA solution, whose results shows that there is no significant response in output voltage. A blank control experiment was also performed by adding PBS buffer and the measured result shows that no interesting response was induced. Both of these control cases indicated that the increase response for 100 ng/mL BSA is caused by the physical adsorption of molecules on the micro-cantilever surface. Figure 7b shows the steady-state responses of the immunosensors versus the concentrations of IgG, which shows a linear relation in the range from 10 ng/mL to 100 ng/mL with the goodness of fit (R^2^-value) above 0.995. 

**Figure 8:**
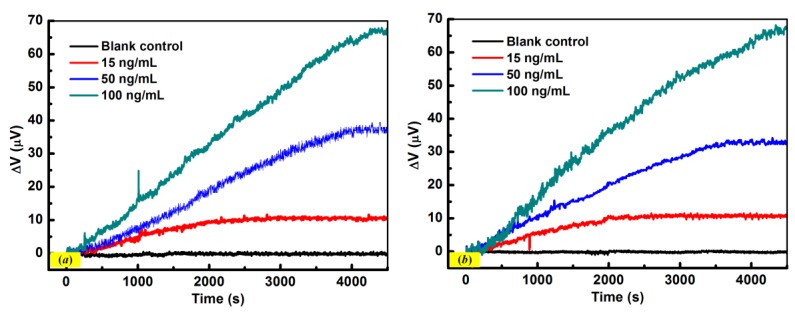
The response curves of immunosensors for the detections of IgG (15, 50, and 100 ng/mL) in (**a**) 0.02 M PBS solution with pH 7.4 and (**b**) 0.01 M PBS solution with neutral pH.

To further evaluate the performance of the proposed polymeric immunosensors, the experiments were performed by changing the ionic strength and pH of PBS solution with identical functionalization procedures. For the experiments in ionic strength, 0.02 M PBS (pH 7.4) was used as buffer solution and the detections of IgG (15, 50, and 100 ng/mL) were performed. The experimental results are shown in Figure 8a, where there is no significant variation for the response signal compared with the measured results by using the 0.01 M PBS solution (pH 7.4). A similar experimental result was also observed when the immunosensors were put in 0.01 M PBS solution with neutral pH for IgG detections with concentrations of 15, 50, and 100 ng/mL (Figure 8b). Therefore, the proposed immunosensors are not sensitive to slight changes in ionic strength or pH. These experimental results indicate that the proposed fully polymeric micro-cantilever based immunosensors could be a potential tool with high selectivity for biological and chemical detection.

## 5. Conclusions

This paper develops a fully polymeric flexible cantilever-based immunosensor for sensitive and selective detection of human IgG in PBS buffer, where PEDOT/PSS conductive layer was fabricated and patterned to be piezoresistors encapsulated in the top and bottom parylene-C layers. The fabricated polymeric micro-cantilever based on PEDOT/PSS conductive layer was demonstrated to have smaller stress mismatch and stiffness compared with the silicon-based cantilever of our previous work [39]. By functionalizing the fabricated cantilever-based sensors, the immunoassay was realized for the detection of human IgG at the concentrations of 10~100 ng/mL, selectively. The immunosensor response exhibited good linearity with a LOD of 10 ng/mL. The proposed flexible polymeric cantilever-based immunosensors promisingly offer a microminiaturization sensing platform for on-site detection.
